# The Relevance of Sexual Dysfunction Related to Groin Pain After Inguinal Hernia Repair – The SexIHQ Short Form Questionnaire Assessment

**DOI:** 10.3389/fsurg.2018.00015

**Published:** 2018-03-19

**Authors:** Nihad Gutlic, Ulf Petersson, Peder Rogmark, Agneta Montgomery

**Affiliations:** ^1^Department of Surgery, Skåne University Hospital, University of Lund, Malmö, Sweden

**Keywords:** inguinal hernia, TEP, sexual dysfunction, quality of life, register, questionnaire

## Abstract

**Background:**

Chronic postoperative pain after inguinal hernia surgery can affect sexual function. A new short form questionnaire for inguinal hernia pain related sexual dysfunction (SexIHQ) was introduced and applied to a register based cohort of total extra-peritoneal hernioplasty (TEP) operated patients.

**Methods:**

Sexually active men, 30–60 years old, recorded in the Swedish Hernia Register for a primary inguinal hernia TEP operation were included. Two initial questions of the SexIHQ identify patients with pain at sexual activity. Only these patients proceeded to answer the specific questions on pain-induced impairment of sexual activity, pain frequency and intensity, physical functions (erection and ejaculation), and symptoms of depression. SexIHQ, the Short Form-36 (SF-36), the Inguinal Pain Questionnaire (IPQ) were mailed to participants for long term follow up.

**Results:**

In 538 included patients, 44 (8.2%) reported pain during sexual activity at mean 33 months after surgery. Sexual dysfunction was seen in 33 of these patients. A postoperative complication was a risk factor for pain during sexual activity; OR 4.89 (95% CI 1.92–12.43; *p* < 0.001). Quality-of-life was reduced in almost all SF-36 domains in patients with pain during sexual activity.

**Conclusions:**

A short form questionnaire, suitable for large cohorts, was developed to assess sexual dysfunction due to groin pain after inguinal hernia repair in male patients. Sexual dysfunction due to groin pain after hernia surgery by TEP is surprisingly common. Patients should preoperatively be informed of the risk of having pain during sexual activity following groin hernia surgery.

## Introduction

Chronic pain as a result of hernia surgery is well-recognized. Knowledge of impairment of sexual function due to pain caused by an inguinal hernia or a postoperative chronic pain condition is, however, limited. Pain affecting sexual function after hernia surgery is believed to be caused by surgical nerve injury, and inflammatory conditions around the mesh or its fixation (1). It can lead to disabling problems, causing impaired sexual function and quality-of-life. Results between studies are difficult to compare since definition of pain and impairment of sexual function varies.

Pre- and postoperative pain-induced sexual dysfunction related to groin hernias has been reported in one retrospective, three prospective, and one randomized controlled trial (RCT) (2–5). The preoperative incidence of sexual dysfunction varies between 10 and 23%, indicating a multitude of instruments being used to assess sexual dysfunction (2, 3, 5, 6). All studies reported diminished sexual dysfunction related to pain after surgery between 4.5 and 10%; with lower rates reported after endoscopic or the Onstep repair compared to the Lichtenstein repair (2, 3, 5, 6).

Two large Danish register-based studies have reported on postoperative complaints. The first assessed 700 patients, of which 72% had been operated on with a Lichtenstein repair. Pain during sexual activity was found in 22%; 6.7% being moderate–severe. A total of 12% had ejaculatory pain (1). The second study reported on 805 TAPP-operated patients where 11% reported pain during sexual activity; 2.4% being moderate–severe pain (7).

Several questionnaires have been developed that specifically address impairment of sexual function. Most are extensive, which may limit the response rate. One example is the validated International Index of Erectile Dysfunction (IIEF) (8). It was used in 40 Lichtenstein-operated patients before and 3 months after surgery, without detected difference (9). The Male Sexual Health Questionnaire (MSHQ) focuses specifically on erectile and ejaculatory dysfunction, as well as intercourse satisfaction (8, 10). These questionnaires do not address the influence of the hernia or sequelae of the operation on sexual dysfunction. A less extensive questionnaire, specifically investigating pain and sexual dysfunction in hernia patients, was developed in Denmark (1). It includes questions on frequency, localization, intensity, pain descriptors, sexual dysfunction due to pain, and pain elsewhere in the body. Although less extensive, this questionnaire would be hard to implement in routine use in large cohorts of patients, as in national registers.

The aim was to introduce a new questionnaire, easy to use for large cohorts of patients, for inguinal hernia-related or postoperative pain-induced sexual dysfunction. The intention was to reduce handling of data by identifying and approaching only patients who have pain at sexual activity and sexual dysfunction related to the groin hernia. The questionnaire was used in a register based study of TEP-operated patients for postoperative assessment of sexual dysfunction.

## Methods

**Figure 1:**
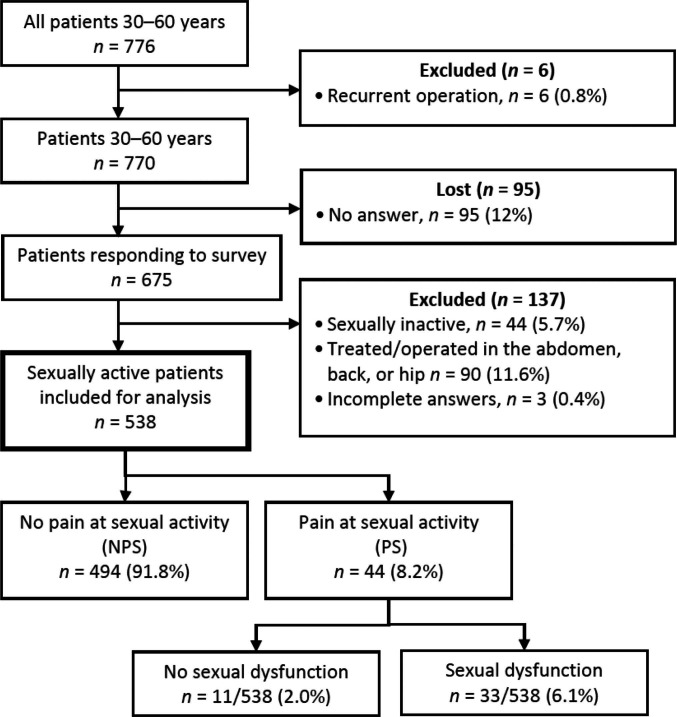
Flowchart.

Men aged 30 to 60 operated on for a primary inguinal hernia by TEP between January 2005 and May 2009 were included (Figure 1). Prospective data from Swedish Hernia Register (SHR) (11) was collected and patients were asked to answer the Sexual Inguinal Hernia Questionnaire (SexIHQ) on pain during sexual activity, the Inguinal Pain Questionnaire IPQ, and the Short Form-36 (SF-36). Inclusions and exclusions are reported in Figure 1. Deceased patients were identified by the Cause of Death Register of the Swedish National Board of Health and Welfare. Patients with previous operations in the abdomen, chronic back or hip pain, or a recurrent hernia were excluded. These are conditions that might have an influence on pain and sexual function that cannot reliably be separated from pain originating from the operated groin. The SHR was searched a second time to identify a recurrent hernia operation during the study period, which was a criterion for exclusion. Questionnaires were mailed in January 2010, followed by one reminder in May, resulting in a minimum follow up of six months.

The trial was approved by the Regional Ethics Review Board at Lund University (634/2008) and registered at www.ClinicalTrials.gov (ID: NCT02419950). A written consent was signed by all participating patients in accordance with the Declaration of Helsinki.

### Questionnaire on Pain During Sexual Activity

**Figure 2:**
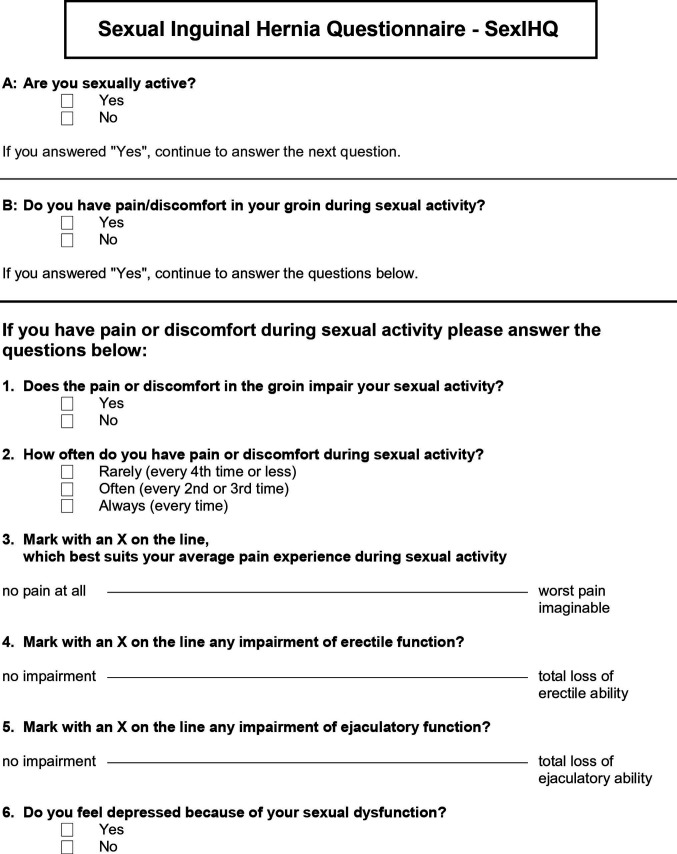
SexIHQ Questionnaire on pain during sexual activity.

The questionnaire was developed by a consensus group of hernia specialists. The purpose was to design a questionnaire specifically addressing pain during sexual activity associated with inguinal hernia surgery, along with sexual dysfunction associated with pain, based on the work by Aasvang et al. (1). The intention was to address modalities of pain as in the original publication, making the questions short, easy and user-friendly. Two initial questions were added to minimize the number of patients having to complete the whole questionnaire. The first question identified “sexually active” patients. The second identified patients having *No pain during sexual activity* (NPS) or *Pain during sexual activity* (PS). There were no further definition given on these questions in the questionnaire. Only patients with *Pain during sexual activity* proceeded to the following questions; assessing pain-induced sexual activity impairment, pain frequency and intensity, physical functions (erection and ejaculation) and symptoms of depression. Out-clinic patients provided feedback on content of the questionnaire, which was adapted to include eight questions forming the SexIHQ (Figure 2).

### The Swedish Hernia Register (SHR)

The SHR is a validated voluntary nationwide prospective register that covers more than 95% of all groin hernia repairs performed in Sweden. Patients enter the register at their primary operation. Complications occurring within 30 days are reported. A recurrent operation is recorded as a new entry. All inhabitants in Sweden have a unique personal identity number, which enables follow-up of patients having subsequent hernia surgery performed anywhere in Sweden. External review of data in the SHR is conducted annually.

Data retrieved in 2010 from the SHR included: age; body mass index (BMI); status according to the American Society of Anesthesiologists (ASA); hernia type; hernia size according to the European Hernia Society classification (12); mesh weight, (heavy-weight: over 50 g/m^2^, light-weight: less than 50 g/m^2^); permanent fixation or non-permanent/no fixation, intraoperative complications; operation time (skin to skin); in-hospital or day-care surgery; and postoperative complications within 30 days (hematoma, infection, urinary retention requiring indwelling catheter, severe pain, reoperation, and unspecified complications).

### Inguinal Pain Questionnaire (IPQ)

IPQ is a validated questionnaire and includes 19 questions regarding different pain modalities (11, 13). Questions 2, 11, 12, 14, 16, and 19 were regarded as relevant for this study. Question 2 *Pain past week* was graded on a 7-level scale from *No pain* to *Pain that could not be ignored; prompt medical advice sought*. The definition of pain was grades 3–7; *pain that could not be ignored, but did not interfere with every day activities* and worse. Grade 1–2; *No pain* and *Pain present that could easily be ignored* was defined as no pain.

### The Short Form-36 (SF-36)

The SF-36 for measurement of health-related quality-of-life (HRQL), licensed by the HRQL-group (www.hrql.se) at Gothenburg University, was used. The subscales and composite scores (physical and mental) were calculated according to the SF-36 manual (14). Norm-based scores were calculated using the Swedish age- and gender-specific means and standard deviations. The norm data have a mean of 50 and a SD of 10. A 5-point difference corresponds to an effect size of 0.5 SD and can be regarded as a medium size clinically important difference (15).

### Statistical Analysis

The IBM SPSS Statistics Software version 22 was used for all statistical analyses. Continuous variables were analyzed using the Student *t*-test, and categorical variables with Pearson *χ*^2^ test or Fisher’s exact test. A *p*-value of less than 0.05 was regarded as significant. Risk factors were selected prior to analysis and entered simultaneously, while odds ratios (OR) were evaluated using binary logistic regression.

## Results

Included patients are reported in a flowchart in Figure 1. 87% of patients (675/776) answered the questionnaire whereof 80% (538/675) met the inclusion criteria and remained for analysis. The follow-up time was 33 (SD 15) months; NPS-patients 33 (SD 15) months, and PS-patients 34 (SD 15) months (*p* < 0.692). The characteristics of the patients are shown in Table 1. Operations were unilateral in 45% and bilateral in 55%.

**Table 1:** Patient characteristics.

	**All patients *n* = 538 (100%)**			**Patients with pain at sexual activity (PS) *n* = 44 (8%)**		
	No pain at sexual activity (NPS)*n* = 494 (92%)	Pain at sexual activity (PS) *n* = 44 (8%)	*****p*-value	No sexual dysfunction *n* = 11 (25%)	Sexual dysfunction*n* = 33 (75%)	***p*-value**
Age [mean (SD)]	51 (8)	48 (8.8)	<0.063	46 (8.4)	49 (8.7)	<0.262
BMI [mean (SD)]	25 (2.4)	26 (3.7)	<0.081	25 (3.0)	26 (3.9)	<0.523
ASA						
ASA I	416 (84)	34 (77)	<0.233	9 (82)	25 (76)	<0.678
ASA II–III	78 (16)	10 (23)	2 (18)		8 (24)	
Hernia type						
Medial	192 (39)	14 (32)	<0.486^*^	2 (18)	12 (36)	<0.240^*^
Lateral	259 (52)	2 (61)		9 (82)	18 (55)	
Femoral	14 (3)	0 (0)		0 (0)	0 (0)	
Combined	29 (6)	3 (7)		0 (0)	3 (9)	
Defect size						
≤3 cm	390 (81)	34 (79)	<0.790	9 (90)	25 (76)	<0.332
>3 cm	93 (19)	9 (21)		1 (10)	8 (24)	
Hernia side						
Unilateral	222 (45)	22 (50)	<0.518	9 (82)	13 (39)	**<0.015**
Bilateral	272 (55)	22 (50)		2 (18)	20 (61)	

^*^Medial vs non-medial (i.e., lateral, femoral or combined).

Percentage within parenthesis unless otherwise stated.

### Pain During Sexual Activity

Operative and postoperative data are shown in Table 2. There were no differences between the NPS and PS groups regarding mesh-weight, fixation, uni- or bilateral operation, intraoperative complications, operation time, and hospital stay. Heavy-weight meshes were used in 66% of patients without difference between the NPS and the PS groups (*p* < 0.364). Immediate severe postoperative pain was seen in 0.6% (3 patients), all in the NPS group. Postoperative complications were reported in a total of 6.5%. Within the NPS 6% reported any complication, and in in the PS group 18% (*p* < 0.005). Postoperative infection was reported in 0.6% (3 patients), all in the NPS group. No reoperation within 30 days was reported.

**Table 2:** Operative and postoperative data.

	All patients *n* = 538 (100%)			Patients with pain at sexual activity PS﻿*n* = 44 (8%)		
	No pain at sexual activity (NPS)*n* = 494 (92%)	Pain at sexual activity (PS)*n* = 44 (8%)	*p*-value	No sexual dysfunction *n* = 11 (25%)	Sexual dysfunction*n* = 33 (75%)	*p*-value
Intra-operative complications						
No	491 (99)	44 (100)	<0.604	11 (100)	33 (100)	<1.000
Yes *(only minor occurred)*	3 (1)	0 (0)		0 (0)	0 (0)	
Operation time, min [mean (SD)]	46 (20)	41 (16)	<0.161	39 (13)	42 (17)	<0.598
Postoperative complications						
No complication	463 (94)	35 (81)		10 (91)	25 (78)	
Any complication	27 (6)	8 (18)		1 (9)	6 (21)	
*Multiple complications*	*1 (0)*	*0 (0)*	<0.005^*^	*0 (0)*	*0 (0)*	<0.673^*^
*Hematoma*	*10 (2)*	*3 (7)*		*0 (0)*	*3 (9)*	
*Infection*	*3 (1)*	*0 (0)*		*0 (0)*	*0 (0)*	
*Severe pain*	*3 (1)*	*0 (0)*		*0 (0)*	*0 (0)*	
*Urinary retention*	*3 (1)*	*1 (2)*		*0 (0)*	*1 (3)*	
*Other (recurrence excluded)*	*7 (1)*	*4 (9)*		*1 (9)*	*3 (9)*	
IPQ 2, pain past week						
No	462 (95)	24 (56)	**<0.001**	6 (55)	18 (56)	<0.922
Yes	24 (5)	19 (44)		5 (45)	14 (44)	
IPQ 11, pain during sports^*^						
No	43 (75)	12 (46)	**<0.009**	6 (75)	6 (33)	**<0.049**
Yes	14 (25)	14 (54)		2 (25)	12 (67)	
IPQ 12, use of analgesics^*^						
No	72 (100)	25 (86)	**<0.001**	9 (100)	16 (80)	<0.148
Yes	0 (0)	4 (14)		0 (0)	4 (20)	
IPQ 14, operation satisfaction						
No	12 (3)	14 (33)	**<0.001**	2 (18)	12 (39)	<0.215
Yes	453 (97)	28 (67)		9 (82)	19 (61)	
IPQ 16, testicular pain						
No	403 (87)	22 (51)	**<0.001**	5 (45)	17 (53)	<0.661
Yes	62 (13)	21 (49)		6 (55)	15 (47)	
IPQ 19, regretting operation						
No	464 (99)	37 (86)	**<0.001**	11 (100)	26 (81)	<0.122
Yes	3 (1)	6 (14)		0 (0)	6 (19)	

^*^Any postoperative complication vs no complication

^†^Question only answered by patients who felt any pain past week (IPQ question 2, level 3–7) (*n* = 101).

Percentage within parenthesis unless otherwise stated.

Significant p values in bold.

**Figure 3:**
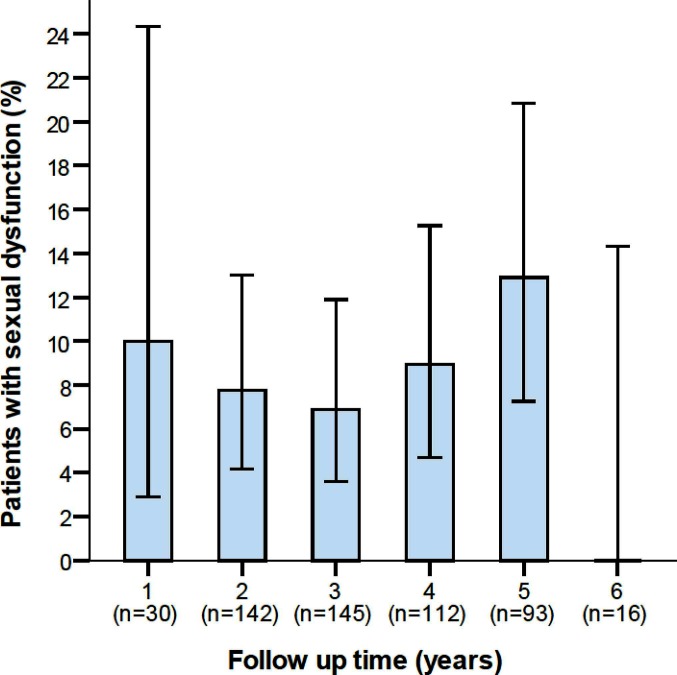
Proportion of patients with sexual dysfunction due to pain in relation to the length of follow-up. Error bars represents 95% confidence intervals.

Results on SexIHQ are reported in Table 3. The option “Always having pain during sexual activity” was reported by 1.5% of the patients, “severe pain” (VAS ≥ 7) by 0.7%, “severe erectile dysfunction” (VAS ≥ 7) by 0.7%, “severe ejaculatory dysfunction” (VAS ≥ 7) by 1.5% and “depression due to sexual dysfunction” by 3.5%. The proportion of patients having “Pain at sexual activity” (PS) did not change over time, Figure 3.

**Table 3:** SexIHQ results (*n* = 44).

**Questions**	**Patients*n* (%)**
1. Impairment of sexual function	
No	11 (32)
Yes	33 (68)
2. Pain frequency	
Rarely	25 (57)
Often	11 (25)
Always	8 (18)
3. Pain (VAS, cm)	
<3	25 (58)
≥3–7	14 (33)
>7	4 (9)
4. Erectile dysfunction (VAS, cm)	
<3	29 (71)
≥3–7	8 (19)
>7	4 (10)
5. Ejaculatory dysfunction (VAS, cm)	
<3	26 (63)
≥3–7	7 (17)
>7	8 (20)
6. Depression	
No	21 (52)
Yes	19 (48)

### Inguinal Pain Questionnaire (IPQ)

The IPQ results are reported in Table 2. Regarding IPQ 2, 8.1% of patients reported chronic pain (levels 3–7), with a difference between the NPS (5%) and PS (44%) groups (*p* < 0.001). Testicular pain, IPQ 16, was reported in total by 16.3% (83/508), with 13% in the NPS and 49% in the PS groups (*p* < 0.001). Overall, 7.1% of patients were unsatisfied and regretted having had the operation: 1% of patients in the NPS and 14% in the PS groups (*p* < 0.001).

### SF-36

**Figure 4:**
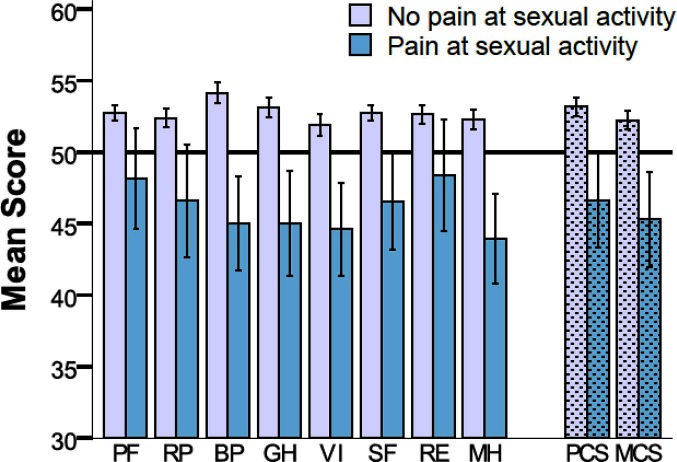
SF-36 shown with norm based scores for eight subscales and two composite scores for patients without (*n* = 494) and with (*n* = 44) pain during sexual activity (mean 50, SD 10). PF, Physical Function; RP, Role Physical; BP, Bodily Pain; GH, General Health; VI, Vitality; SF, Social Function; RE, Role Emotional; MH, Mental Health; and PCS, Physical Composite Score; MCS, Mental Composite Score. Error bars represents 95% confidence intervals.

All subscales for SF-36 were slightly above the norm in NPS patients, Figure 4. In PS patients, all SF-36 scales were significantly lower compared to the norm, except for Physical Function (PF), Role Physical (RP), and Role Emotional (RE).

### Risk Factor Analysis

**Figure 5:**
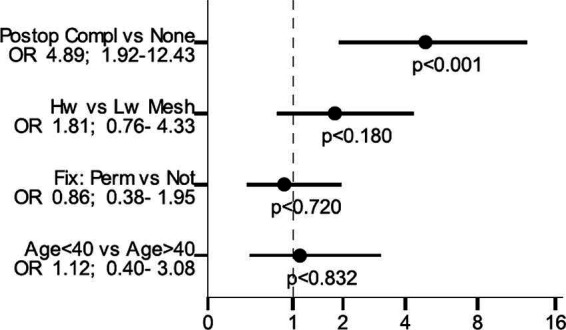
Risk factor analysis for sexual dysfunction related to pain.

Risk factor analysis for sexual dysfunction related to pain is presented in Figure 5. A postoperative complication was the only independent risk factor for sexual dysfunction due to pain in a multivariable model: OR 4.89 (95% CI 1.92–12.43; *p* < 0.001).

## Discussion

This study is based on the Swedish National Hernia Register, introducing the SexIHQ questionnaire specifically addressing impairment of sexual function related to pain after inguinal hernia repair with TEP. Long-term sexual impairment after groin hernia operation was present in one out of twelve patients and did not seem to attenuate over time. Quality-of-life was considerably reduced in afflicted patients.

A relatively short questionnaire, specifically investigating genital pain and sexual dysfunction in hernia patients was developed by Aasvang et al. (1). This protocol was further developed and used in a more detailed protocol (7). We enhanced these ideas with the purpose of making a more specific questionnaire, the SexIHQ, which only assesses impairment of sexual function caused by pain after surgical repair. The debilitating effect of pain on both erectile and ejaculatory function, as well as psychological problems (e.g., depression), seemed relevant to include as a dimension in the quality-of-life concept. This is supported by the decreased dimensions of SF-36 in both physical and mental domains. To assess sexual dysfunction, visual analogue scales and tick-boxes were used in this 8-question 1-page questionnaire, which was created for implementation in large cohort studies (e.g., register-based). The goal was to approach only patients having sexual impairment after hernia repair. For this reason, the questionnaire starts with two questions discriminating sexually active from sexually inactive patients, defining sexually active patients as having pain during sexual activity versus those without. This method limits the number of patients needed to be addressed with more specific questions on sexual issues, hopefully increasing the response rate in large cohorts.

A limitation of this study is the lack of preoperative data. These data are not available in most register-based studies today. Even if all questions focus on the relation between “pain in the groin” and sexual function, it could still be difficult for the patient to differentiate between postoperative groin pain and other conditions as a cause for the impairment of sexual function. In a recently published RCT (Lichtenstein versus the Onstep technique), 28% of patients reported pain during sexual activity preoperatively, but reduced to 11% postoperatively (5). In another study on TAPP, the corresponding values were 23 and 10% (4). Our results, in a national cohort of patients operated on by several different surgeons at different educational levels, experienced 8.2% of pain during sexual activity. Among these patients 6.1% (33/44) reported sexual dysfunction; this indicates that the TEP repair may be a competitive technique in this perspective.

Including patients from a national register has its advantages. All levels of experience and varying techniques used by operating surgeons are included, resulting in high external validity. Three studies on this subject were published from the Danish national hernia register (1, 4, 7).

A register-based questionnaire study of 1,172 patients operated on by TAPP during a 10 year period had a response rate of 68% (7). We achieved a response rate of 86% in patients operated on during a 5 year period, which is regarded as excellent. One explanation might be that our questionnaire was limited to very few questions, whereas only pain-afflicted patients (8%) were requested to answer them.

The only risk factor for impairment of sexual function was a postoperative complication. Preoperative pain has also been demonstrated to be a risk factor for postoperative pain (4). We were unable to evaluate this finding as no preoperative pain status is recorded in the SHR.

## Conclusion

We present a short form questionnaire for cohorts that assesses sexual dysfunction related to chronic postoperative pain in a national cohort of TEP-operated men. Care should be taken to prevent complications, thereby reducing the risk of sexual dysfunction. Patients should be preoperatively informed about the risk of pain, and also about the risk of sexual dysfunction after groin hernia surgery.

## Ethics Statement

The trial was approved by the Regional Ethics Review Board at Lund University (634/2008) and registered at www.ClinicalTrials.gov (ID: NCT02419950). A written consent was signed by all participating patients in accordance with the Declaration of Helsinki.

## Author Contributions

NG has been engaged in all the parts of the study including planning, sending all questionnaires, creating the data platform, analyzing data, performing statistical calculations, preparing tables, figures and the manuscript. PR has been engaged in all the parts of the study, preparing the data base platform, double-checking all statistical calculations, preparing the manuscript, tables and figures, and revising the manuscript. UP been engaged in all the parts of the study, analyzing data and preparing the manuscript. AM, being the principle investigator of the project, has been engaged in all the parts of the study, project planning, applications, data analysis, interpretation of data and preparing the manuscript.

## Conflict of Interest Statement

The authors declare that the research was conducted in the absence of any commercial or financial relationships that could be construed as a potential conflict of interest.

## References

[1] Aasvang EK Møhl B Bay-Nielsen M Kehlet H Pain related sexual dysfunction after inguinal herniorrhaphy. *Pain* (2006) 122(3):258–63. 10.1016/j.pain.2006.01.035 16545910

[2] Zieren J Menenakos C Paul M Müller JM Sexual function before and after mesh repair of inguinal hernia. *Int J Urol* (2005) 12(1):35–8. 10.1111/j.1442-2042.2004.00983.x 15661052

[3] Bittner R Gmähle E Gmähle B Schwarz J Aasvang E Kehlet H Lightweight mesh and noninvasive fixation: an effective concept for prevention of chronic pain with laparoscopic hernia repair (TAPP). *Surg Endosc* (2010) 24(12):2958–64. 10.1007/s00464-010-1140-9 20526620

[4] Tolver MA Rosenberg J Pain during sexual activity before and after laparoscopic inguinal hernia repair. *Surg Endosc* (2015) 29(12):3722–5. 10.1007/s00464-015-4143-8 25783834

[5] Andresen K Burcharth J Fonnes S Hupfeld L Rothman JP Deigaard S Sexual dysfunction after inguinal hernia repair with the Onstep versus Lichtenstein technique: a randomized clinical trial. *Surgery* (2017) 161(6):1690–5. 10.1016/j.surg.2016.12.030 28262253

[6] Schouten N van Dalen T Smakman N Clevers GJ Davids PH Verleisdonk EJ Impairment of sexual activity before and after endoscopic totally extraperitoneal (TEP) hernia repair. *Surg Endosc* (2012) 26(1):230–4. 10.1007/s00464-011-1859-y 21959685

[7] Bischoff JM Linderoth G Aasvang EK Werner MU Kehlet H Dysejaculation after laparoscopic inguinal herniorrhaphy: a nationwide questionnaire study. *Surg Endosc* (2012) 26(4):979–83. 10.1007/s00464-011-1980-y 22011952

[8] Rosen RC Catania JA Althof SE Pollack LM O'Leary M Seftel AD Development and validation of four-item version of male sexual health questionnaire to assess ejaculatory dysfunction. *Urology* (2007) 69(5):805–9. 10.1016/j.urology.2007.02.036 17482908

[9] Bulus H Dogan M Tas A Agladıoglu K Coskun A The effects of Lichtenstein tension-free mesh hernia repair on testicular arterial perfusion and sexual functions. *Wien Klin Wochenschr* (2013) 125(3-4):96–9. 10.1007/s00508-013-0321-7 23334479

[10] Rosen RC Catania J Pollack L Althof S O'Leary M Seftel AD Male Sexual Health Questionnaire (MSHQ): scale development and psychometric validation. *Urology* (2004) 64(4):777–82. 10.1016/j.urology.2004.04.056 15491719

[11] Gutlic N Rogmark P Nordin P Petersson U Montgomery A Impact of mesh fixation on chronic pain in total extraperitoneal inguinal hernia repair (TEP): a nationwide register-based study. *Ann Surg* (2015). 10.1097/SLA.0000000000001306 26135697

[12] Miserez M Alexandre JH Campanelli G Corcione F Cuccurullo D Pascual MH The European hernia society groin hernia classification: simple and easy to remember. *Hernia* (2007) 11(2):113–6. 10.1007/s10029-007-0198-3 17353992

[13] Fränneby U Gunnarsson U Andersson M Heuman R Nordin P Nyrén O Validation of an inguinal pain questionnaire for assessment of chronic pain after groin hernia repair. *Br J Surg* (2008) 95(4):488–93. 10.1002/bjs.6014 18161900

[14] Sullivan M Karlsson J Taft C Ware JE *SF-36 Hälsoenkät: Svensk Manual och Tolkningsguide, 2:a upplagan [Swedish Manual and Interpretation Guide]*. 2nd ed Gothenburg: Sahlgrenska University Hospital (2002).

[15] Cohen J *Statistical power analysis for the behavioral sciences*. Hillsdale, NJL: Erlbaum Associates (1988).

